# Case Report: Prenatal Whole-Exome Sequencing to Identify a Novel Heterozygous Synonymous Variant in NIPBL in a Fetus With Cornelia de Lange Syndrome

**DOI:** 10.3389/fgene.2021.628890

**Published:** 2021-02-09

**Authors:** Fengchang Qiao, Cuiping Zhang, Yan Wang, Gang Liu, Binbin Shao, Ping Hu, Zhengfeng Xu

**Affiliations:** Department of Prenatal Diagnosis, Nanjing Maternity and Child Health Care Hospital, Women's Hospital of Nanjing Medical University, Nanjing, China

**Keywords:** Cornelia de Lange syndrome, whole-exome sequencing, NIPBL, splicing mutation, prenatal diagnosis

## Abstract

Cornelia de Lange syndrome (CdLS) is a genetically heterogeneous disorder characterized by a wide spectrum of abnormalities, including craniofacial dysmorphism, upper limb anomalies, pre- and post-natal growth restrictions, hirsutism and intellectual disability. Approximately 60% of cases are caused by *NIPBL* variants. Herein we report on a prenatal case presented with bilateral upper-extremity malformations and cardiac defects. Whole-exome sequencing (WES) was performed on the fetus–parental trio and a *de novo* heterozygous synonymous variant in *NIPBL* [chr5:37020979; NM_133433.4: c.5328G>A, p. (Gln1776=)] was identified. Reverse transcriptase–polymerase chain reaction (RT–PCR) was conducted to evaluate the potential splicing effect of this variant, which confirmed that the variant caused a deletion of exon 27 (103 bp) by disrupting the splice-donor site and changed the reading frame with the insertion of at least three stop codons. Our finding not only expands the mutation spectrum of *NIPBL* gene but also establishes the crucial role of WES in searching for underlying genetic variants. In addition, our research raises the important issue that synonymous mutations may be potential pathogenic variants and should not be neglected in clinical diagnoses.

## Introduction

Cornelia de Lange syndrome (CdLS, MIM #122470, #300590, #610759, #614701, and #300882), a congenital developmental disorder inherited in an autosomal dominant (*NIPBL, SMC3*, and *RAD21*) or X-linked (*SMC1A* and *HDAC8*) manner, was initially discovered by Brachmann (1916) and was further summarized by de Lange (1933) (Jang et al., 2015; Li et al., 2020). CdLS is complicated by a wide spectrum of abnormalities, including craniofacial dysmorphism, upper limb anomalies, pre- and post-natal growth restrictions, hirsutism, and intellectual disability (Lalatta et al., 2007). Facial dysmorphisms include micrognathia, synophrys, long eyelashes, depressed nasal bridge, low-set ears, small head, small and widely spaced teeth, long philtrum and thin lips (Ireland and Burn, 1993). CdLS occurs sporadically with a prevalence of 1 in 10,000 to 1 in 30,000 live births (Kline et al., 2018); however, the actual number is far more than that as some mild cases with atypical symptoms are not clinically diagnosed.

CdLS is typically diagnosed after birth based on some abnormal phenotypes (Kinjo et al., 2019). Although several cases have been reported before birth, prenatal diagnosis is still a challenge for clinicians and geneticists, with only 23% of all cases being detected prenatally (Avagliano et al., 2017). It has been reported that maternal serum pregnancy-associated placental protein-A (PAPP-A) produced by the placenta in the first and second trimester of pregnancy is an early indicator of CdLS, but it is not specific as only a very low level can support this diagnosis (Kinjo et al., 2019). At present, prenatal diagnosis of CdLS primarily relies on sonographic findings during the second and third trimester of pregnancy, such as characteristic facial features and congenital malformations. However, some such prenatal phenotypes that are detected by ultrasound are not unique to CdLS. Undoubtedly, the complexity and variability of CdLS phenotypes increase the difficulty of prenatal diagnosis. Therefore, considerable clinical experience and professional knowledge about the dysmorphic features of CdLS are still needed for clinicians.

With the rapid development of molecular diagnostic technology, the variants of relative pathogenic genes can be confirmed by molecular testing, which is the key to diagnosis. To date, it has been acknowledged that pathogenic variants of five genes encoding the structural components (*SMC1A, SMC3*, and *RAD21*) or function-related factors (*NIPBL* and *HDAC8*) of the cohesin complex are associated with CdLS. In particular, the heterozygous mutations of *NIPBL* account for ~60–70% of all CdLS cases (Teresa-Rodrigo et al., 2014; Kline et al., 2018). *NIPBL* localized on 5p13.2 facilitates cohesin loading on chromatin, whereas the cohesin complex is involved in sister chromatid cohesion and ensures accurate chromosome segregation, recombination-mediated DNA repair, and genomic stability (Baquero-Montoya et al., 2014; Teresa-Rodrigo et al., 2014). It has been reported that the variants in *NIPBL* also manifest as severe CdLS phenotypes with a high frequency of limb anomalies (Selicorni et al., 2007). Additionally, individuals with a somatic heterozygous pathogenic mutation in *NIPBL* appear to be fully penetrant (Deardorff et al., 1993). Nevertheless, for 20–30% of affected individuals, molecular alterations cannot be found in the aforementioned genes (Pie et al., 2016; Wagner et al., 2019).

Whole-exome sequencing (WES), especially for the fetus–parental trio, has proven to be a feasible and effective approach for the identification of some rare disorders that are suggestive of genetic etiology but cannot be diagnosed using conventional testing methods. In two large prospective cohort studies, Petrovski and Lord and their respective teams identified an additional 8.5 and 10% diagnostic genetic variants, respectively, through WES for cases that tested negative in karyotype and chromosomal microarray analyses (CMA), which further confirmed the feasibility and potential value of prenatal WES in detecting genetic diseases (Lord et al., 2019; Petrovski et al., 2019). Currently, WES is a first-line detection method for hereditary diseases. In our study, we detected a *de novo* heterozygous variant of *NIPBL* in a fetus with bilateral upper-extremity malformations and cardiac defects via the application of WES, which provides a theoretical basis and guidance for this family in reproductive genetic counseling and prenatal diagnosis.

## Materials and Methods

### Ethics Statement

This research was approved by the Ethics Committee of the Nanjing Maternity and Child Health Care Hospital and adhered to the Declaration of Helsinki. Samples and information were collected from the parents after written informed consent was obtained. In addition, they agreed to the publication of the manuscript.

### Case Report

A 26-year-old pregnant Chinese woman gravida 2 para 0, who was referred to the Center of Prenatal Diagnosis at Nanjing Maternity and Child Health Care Hospital opted for genetic tests because of an ultrasonic abnormality. The woman and her husband were healthy and non-consanguineous, with no history of infection and/or exposure to teratogens. However, their family history was significant for a previous pregnancy that was terminated because of skeletal and cardiac dysplasia.

**Figure 1:**
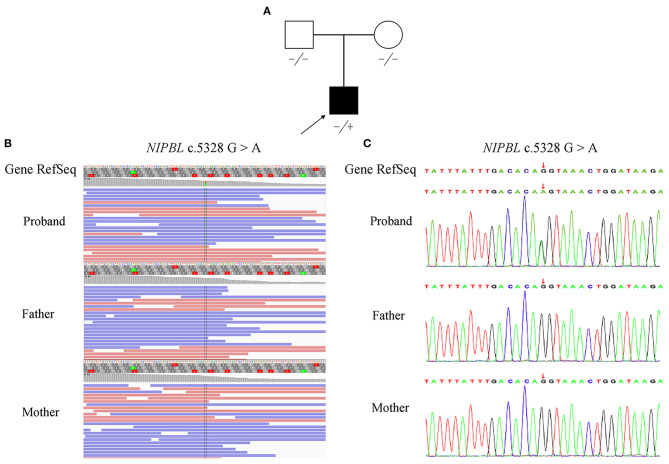
Pedigree and mutation analysis of the family. **(A)** Pedigree of the family with segregation of the identified *NIPBL* mutation. The square represents the male, and circle represents the female. A filled symbol indicates the proband. **(B)** Visualization of the mutation in *NIPBL* is shown with an integrative genomics viewer. The variant (c.5328G>A) was heterozygous in the proband. **(C)** Confirmation of the mutation (c.5328G>A), which was not found in his parents, was conducted by Sanger sequencing.

At 24 weeks of gestation, a prenatal ultrasound examination in our hospital indicated skeletal dysplasia of the bilateral upper limbs and congenital heart malformation. The bilateral humerus was short, ~1.93 cm, and only the ulna was angled with the humerus. Additionally, the fetus had congenital cardiac developmental abnormalities, and the structures of the ventricular outflow tract were unclear because of the small gestational age. After genetic counseling by the clinic's geneticist, the couple ultimately decided to terminate the pregnancy at 27^+3^ weeks because of the fetal malformations and uncertainty of prognosis. However, they did not consent to a pathological autopsy. To assess the risk of recurrence, they requested genetic testing to clarify the potential cause of the disease. Because of the negative results of CMA test, we applied WES for the proband and his healthy parents (Figure 1A) to search for potential variants. The detailed examinations of pregnant woman are listed in Supplementary Table 1.

### Whole-Exome Sequencing

Genomic DNA was extracted from the proband's tissue sample after the pregnancy was terminated and from the parental peripheral blood using an Automated Nucleic Acid Extractor (RBCBioscience) following standard procedures for WES analysis. First, genomic DNA was sheared and amplified to generate a fragment library with a read length of 150 bp that was captured by the IDT The xGen Exome Research Panel v1.0 according to the manufacturer's protocols. Sequencing was subsequently carried out on the Illumina NovaSeq 6000 (Illumina, Inc.) platform, and the resultant reads were mapped against GRCh37/hg19 assembly. Single-nucleotide variants (SNVs) and small indels (<50 bp) were identified using the Genome Analysis Toolkit 3.4 (GATK). Novel mutations were filtered against the 1000 Genomes database (1000 genomes release phase 3, http://www.1000genomes.org/), dbSNP database (http://www.ncbi.nlm.nih.gov/projects/SNP/snp_summary.cgi), and the genome aggregation database (gnomad.broadinstitute.org). Finally, polymerase chain reaction (PCR) was performed to amplify the affected fragment of *NIPBL* using specific primers (forward primer: 5′-TACACCCGGCCGGAGAACCTAAAA-3′ and reverse primer: 5′-ATGCATCGAGCTGAAAACCGAAAA-3′), and the purified PCR products were applied to Sanger sequencing to affirm the mutation.

### RNA Transcript Analysis by RT–PCR

MaxEntScan (Wappenschmidt et al., 2012; Afzal et al., 2019) and dbscSNV (Jian et al., 2014) were used to predict whether the variant affects splice site. To detect aberrant transcripts, total RNA was isolated from fetal skin and control cells using TRIzol™ Reagent (Invitrogen; Thermo Fisher Scientific) according to the manufacturer's protocols. Complementary DNA (cDNA) was synthesized from the total RNA using a PrimeScript™ RT reagent Kit with gDNA Eraser (TAKARA), which was further amplified to obtain PCR products using specific primers (forward primer: 5′-AGGAACACATCATGCAAAGGA-3′ and reverse primer: 5′-ACAGCAACAACCTCAGACAA-3′). PCR products were ultimately separated on a 2% agarose gel and validated by Sanger sequencing.

## Results

We first examined the CMA for the proband, but no chromosomal abnormalities or submicroscopic chromosomal imbalances were detected at the whole genome level. Then, we performed sequencing and subsequent alignment of the whole exome and obtained 77.82 million reads with a read length of 150 bp and average sequencing depth of 99.59 × and 98.93% of the whole exome target region covered at ≥20 ×. There were 32,561 single-nucleotide polymorphisms (SNPs), including 3,432 non-synonymous SNPs and 1,725 synonymous SNPs in the coding sequence, as well as 35 SNPs in the splice sites. In addition, there were 244 indels in the coding sequence.

**Figure 2:**
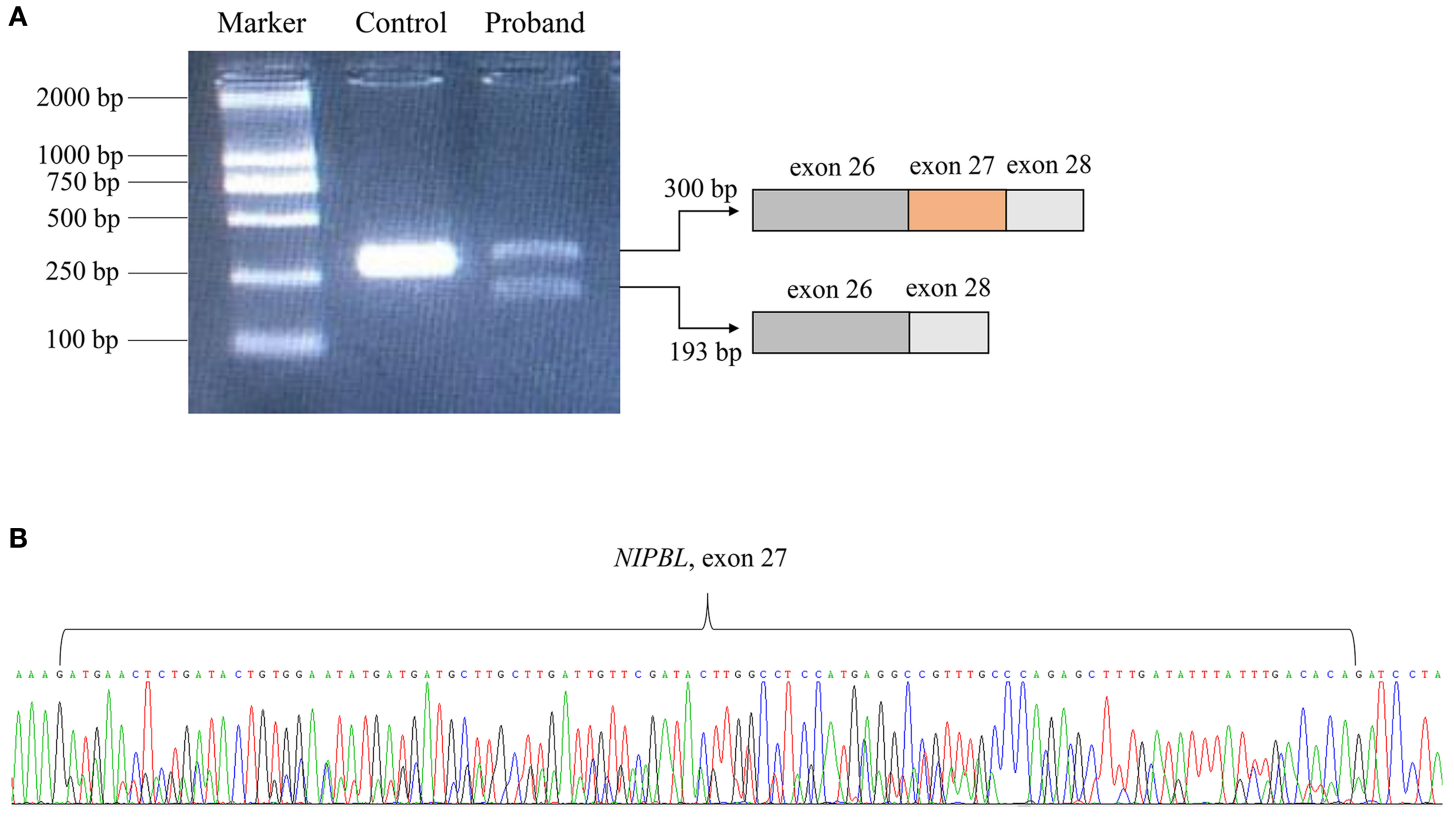
RNA transcript analysis using specific RT-PCR. **(A)** Gel image showing the PCR products of *NIPBL* cDNA fragments. **(B)** The deletion of exon 27 in *NIPBL* mRNA was confirmed by Sanger sequencing.

Following the prioritized filtration strategy (Roy et al., 2018), we selected nine candidate variants, including seven missense mutations and two frameshift mutations (Supplementary Table 2). Notably, a plausible point mutation in *NIPBL* [chr5:37020979; NM_133433.4: c.5328G>A, p. (Gln1776=)] may explain the features described above, considering the inheritance model of the disease and phenotypes of the gene. By bioinformatics, the variant is predicted to affect splicing according to MaxEntScan and dbscSNV (Supplementary Table 3). Next, we performed RNA transcript analysis using specific RT-PCR to explore the implication of this aberrant variant on the splicing of *NIPBL*. The resultant PCR products of the control RNA showed that a single amplified cDNA fragment of 300 bp was expected of a normal transcript, whereas the proband showed two bands comprising an aberrant band (197 bp) that was smaller than that of the control cells (Figure 2A). Sanger sequencing cDNA data analysis revealed that the aberrant transcript was affected by the variant, thus leading to complete skipping of exon 27 (103 bp) (Figure 2B). Although the variant was located at the last nucleotide of exon 27 and not within the canonical splice sites, the variant affects splicing generating an aberrant transcript with complete absence of exon 27 characterized by altered protein reading frame and premature stop codons (ACMG variant evidence PVS1). Using Sanger sequencing, the variant was further verified as a *de novo* heterozygous synonymous mutation in the proband, which means that it was not identified in either of the non-consanguineous parents (ACMG variant evidence PS2) (Figures 1B,C). Furthermore, this variant was absent from the 1000 Genomes, ExAC, or dbSNP databases (ACMG variant evidence PM2). Moreover, the probability of loss-of-function intolerance (pLI) is 1 in the ExAC Browser, which suggests that *NIPBL* is highly intolerant to heterozygous loss-of-function variants. Considered together, the variant (c.5328G>A) was classified as a pathogenic mutation according to the ACMG guidelines (Richards et al., 2015).

In addition, the remaining eight candidate variants were excluded because of insufficient evidence. *COL11A2, DCSH1, POC1A*, and *WDR60* follow the model of autosomal recessive inheritance, but the proband with heterozygosity variants was not affected. Although the variant of *RAB40AL* was assessed as likely pathogenic, its syndrome phenotype was not consistent with the ultrasonic phenotype of the fetus. Furthermore, the mutations of *IFIH1, WHSC1*, and *TBX4* were evaluated as variants of uncertain significance (Supplementary Table 2).

## Discussion

Here, we report a fetus with CdLS whose diagnosis was prompted by abnormal sonographic findings of upper extremity anomalies and congenital heart defects. The cause of the disease was a previously undescribed *de novo* heterozygous synonymous variant in *NIPBL* (c.5328G>A) that was identified by WES. The variant located in the exon–intron border disrupted the normal splicing process as predicted by bioinformatics. By further validation using RT-PCR and Sanger sequencing, we detected the deletion of exon 27 (103 bp, 1,776–1,810 aa) at the mRNA level in only the proband.

*NIPBL* encodes the Nipped-B-like protein (delangin), which plays a pivotal role in loading the cohesin complex onto DNA and is crucial for sister chromatid cohesion and regulation of other proteins (Gause et al., 2008; Bot et al., 2017). To our knowledge, *NIPBL* is expressed in developing limbs and subsequently in cartilage primordia of the ulna and various hand bones, as well as in the developing heart, especially in the atrial and ventricular myocardium during the embryo stage (Ben-Asher and Lancet, 2004; Tonkin et al., 2004; Kang et al., 2018). In the case presented here, a prenatal ultrasound suggested that the bilateral humerus of the fetus was shorter than expected at standard gestational age, and the metacarpal bone could not be clearly identified. Furthermore, the fetus showed congenital cardiac developmental abnormalities. Combining the bias of *NIPBL* expression regions and the phenotypic characteristics of the fetus, we inferred that the mutation of *NIPBL* is correlated with the aberrant fetal phenotypes.

It is universally acknowledged that synonymous mutations are often neglected as silent variants, which may be because of a lack of alteration in the composition of amino acids caused by the degeneracy of the genetic code. To narrow down candidate mutations, synonymous mutations are usually eliminated first as benign variants in clinical analysis. However, with the advancement of research, mounting evidence illustrates the pathogenic mechanism of synonymous mutation by affecting the translation rate, mRNA stability, protein folding, and splicing accuracy in human diseases (Cartegni et al., 2002; Nackley et al., 2006; Sauna and Kimchi-Sarfaty, 2011). Previous studies have demonstrated that synonymous mutations could affect the splicing accuracy. According to a study by Faa et al. (2010), a synonymous mutation (c.2811G>T) in *CFTR* of an Italian patient with cystic fibrosis created a new 5′ splice site within exon 15, which generated a transcript lacking 76 amino acid residues. The aberrant protein may affect the structure and regulation of the chloride (Cl^−^) channel (Faa et al., 2010). Furthermore, Kim et al. (2013) also reported a novel synonymous mutation (c.1803G>A) in *SLC26A4* that resulted in complete skipping of exon 16 by affecting the splicing accuracy in two members of a Korean family suffering from hearing impairment (Kim et al., 2013). All these described cases reveal that synonymous mutations should be evaluated separately in clinical diagnoses as they usually affect splicing. As described in our case, the absence of exon 27 was caused by a synonymous mutation, which was suspected to affect the splice-donor site of *NIPBL*. To some degree, exon 27 skipping may impact the patient's phenotypes.

To our knowledge, the most common effect of splicing site changes is skipping of the next exon. In our case, this novel variant c.5328G>A occurred at the terminal nucleotide of exon 27 and generated an aberrant transcript with an exon 27 deletion by affecting the splicing accuracy. Previously, Teresa-Rodrigo et al. (2014) discovered that splice site mutations account for ~17% of *NIPBL* variants. The variant (c.5328+1G>A) in *NIPBL* that they reported was at a different in genomic location from that reported in our case (c.5328G>A), but coincidentally generated an identical splicing effect with the skipping of exon 27. In addition, the patient seemed to have the same severe structural anomalies of the limbs as in our case, such as bilateral aplasia of the ulna and distal humerus (Teresa-Rodrigo et al., 2014). Exon 27 is located in an evolutionarily conserved protein domain called HEAT repeats (H1: 1,767–1,805 aa, H2: 1,843–1,881 aa, H3: 1,945–1,984 aa, H4: 2,227–2,267 aa, H5: 2,313–2,351 aa), which is an essential component of the *NIPBL* gene product (delangin) and function in protein–protein interaction motifs (Neuwald and Hirano, 2000; Mannini et al., 2013; Li et al., 2020). On the one hand, absence of exon 27 might disrupt the integrity of the HEAT repeats. A previous study showed that the C-terminal region containing HEAT repeats and the heterochromatin protein 1 (HP1) interacting motif could recruit *NIPBL* to the sites of DNA damage, thus indicating the HEAT repeats are essential to repair DNA damage (Oka et al., 2011). In another research, Gause et al. (2008) proposed that some of the HEAT repeats are crucial for gene expression and sister chromatid cohesion. On the other hand, exon 27 skipping disrupted the reading frame and inserted premature stop codons that may have resulted in degradation of the corresponding transcript by nonsense-mediated mRNA decay. Consequently, the putative pathogenic mechanism in this case would be haploinsufficiency. Further exploration is needed to attain a detailed understanding of the effects of this variant at the transcript and protein level.

Although the mother of the proband experienced the adverse outcome of terminated pregnancy because of skeletal and cardiac dysplasia in her first pregnancy, we found a *de novo* explicit mutation in *COL1A1* [chr17:48264402; NM_000088.4: c.3505G>A, p. (Gly1169Ser)] in the first fetus by trio WES. Furthermore, the variant was already reported as pathogenic in ClinVar: SCV000695351.1 (Pathogenic^*^-Osteogenesis imperfecta), SCV000883622.1 (Pathogenic^*^-not provided), SCV000955221.1 (Pathogenic^*^-Osteogenesis imperfecta type I), SCV000574580.1 (Pathogenic^*^-Osteogenesis imperfecta type I). Therefore, the different causes of two adverse outcomes contribute to us ruling out the possibility of gonadal mosaicism. For future pregnancy, prenatal diagnosis is necessary for the fetus, especially to detect the above-mentioned mutations in these two genes.

Overall, this study presented the case of a fetus with CdLS with a *de novo* heterozygous mutation (c.5328G>A) in *NIPBL*. The synonymous mutation affects the normal splicing process and produces a frameshift that results in premature stop codons. Thus, the variant identified in our case is valuable for comprehending possible pathogenic mechanisms of *NIPBL*, which not only broadens the mutation spectrum of *NIPBL* but also contributes to supportive genetic counseling and timely management for pregnancies with a structural abnormality. Finally, we cannot ignore novel synonymous variants as silent mutations in clinical analysis; sometimes these variants elucidate the potential pathogenic mechanism of a disease.

## Data Availability Statement

All datasets for this study are included in the article/Supplementary Tables.

## Ethics Statement

This research was approved by the Ethics Committee of the Nanjing Maternity and Child Health Care Hospital and adhered to the Declaration of Helsinki. Samples and information were collected from fetal parents after written informed consent was obtained and they also agreed to the publication of the manuscript.

## Author Contributions

FQ and CZ performed the experiments, wrote the manuscript, and analyzed the WES data. YW and BS collected the clinical information and provided the genetics counseling. GL conducted the bioinformatics analysis. PH and ZX designed the study and revised the manuscript. All authors read and approved the final manuscript.

## Conflict of Interest

The authors declare that the research was conducted in the absence of any commercial or financial relationships that could be construed as a potential conflict of interest.
