# Supplementary material for: Case Report: Prenatal Whole-Exome Sequencing to Identify a Novel Heterozygous Synonymous Variant in NIPBL in a Fetus With Cornelia de Lange Syndrome
Source: Front Genet. 2021 Feb 9;12:628890. doi: 10.3389/fgene.2021.628890 (PMC7900548; doi:10.3389/fgene.2021.628890)
Supplement: Supplementary file 3 [file Table_3.docx]

Table S3. In silico characterization of analyzed variant.

| variant | MaxEntScan | | | dbscSNV | |
| --- | --- | --- | --- | --- | --- |
|  | wild type | mutant | % | ada score | rf score |
| *NIPBL*, c.5328G>A | 7.82 | 1.87 | -76.09 | 0.9999 | 0.994 |
